# Quantitative Structure-Antioxidant Activity Models of Isoflavonoids: A Theoretical Study

**DOI:** 10.3390/ijms160612891

**Published:** 2015-06-08

**Authors:** Gloria Castellano, Francisco Torrens

**Affiliations:** 1 Departamento de Ciencias Experimentales y Matemáticas, Facultad de Veterinaria y Ciencias Experimentales, Universidad Católica de Valencia *San Vicente Mártir*, Guillem de Castro-94, E-46001 València, Spain; 2 Institut Universitari de Ciència Molecular, Universitat de València, Edifici d’Instituts de Paterna, P. O. Box 22085, E-46071 València, Spain; E-Mail: torrens@uv.es

**Keywords:** antioxidant, structure-activity relationship, absorption, QSAR, ADMET, poor absorption or permeation

## Abstract

Seventeen isoflavonoids from isoflavone, isoflavanone and isoflavan classes are selected from *Dalbergia parviflora*. The ChEMBL database is representative from these molecules, most of which result highly drug-like. Binary rules appear risky for the selection of compounds with high antioxidant capacity in complementary xanthine/xanthine oxidase, ORAC, and DPPH model assays. Isoflavonoid structure-activity analysis shows the most important properties (log *P*, log *D*, p*K*_a_, QED, PSA, NH + OH ≈ HBD, N + O ≈ HBA). Some descriptors (PSA, HBD) are detected as more important than others (size measure *M*w, HBA). Linear and nonlinear models of antioxidant potency are obtained. Weak nonlinear relationships appear between log *P*, *etc*. and antioxidant activity. The different capacity trends for the three complementary assays are explained. Isoflavonoids potency depends on the chemical form that determines their solubility. Results from isoflavonoids analysis will be useful for activity prediction of new sets of flavones and to design drugs with antioxidant capacity, which will prove beneficial for health with implications for antiageing therapy.

## 1. Introduction

Flavonoids and isoflavonoids influence intercellular redox status to interact with specific proteins in intracellular signaling pathways and present antioxidant properties [1]. Antioxidants are chemical entities that function breaking free-radical chain reaction and metal ion chelation, which would catalyze free-radical-induced systemic damage. The molecules are polyphenolic and electron-rich, potentially acting as substrate inhibitors for the cytochrome P450 (CYP) enzymes and inducing detoxification enzymes, e.g., CYP-dependent monooxygenases (MOs) [2]. Some polyphenols penetrate the blood-brain barrier (BBB) into regions mediating cognitive behavior [3]. Because of flavonoids structural diversity, quantitative structure-activity relationships (SARs) (QSARs) were studied via antioxidant capacity assays [4]. Flavonoids potency depends on their chemical structure, which is influenced by the number and position of hydroxyl groups (OH) attached to both aromatic rings [5]. Isoflavonoids QSARs are scarce [6,7,8,9]. Isoflavonoids antioxidant activity depends on the redox properties of their hydroxyphenolic groups and structural relationship among the different moieties of the chemical structure, which allows many substitution patterns and variations on ring C (Table 1). Promden *et al*. evaluated antioxidant activities of 24 isoflavonoids from *Dalbergia parviflora* via three complementary *in vitro* antioxidant-based assay systems [10]: xanthine/xanthine oxidase (X/XO) [11], oxygen radical absorbance capacity (ORAC) [12] and 2,2-diphenyl-1-picrylhydrazyl (DPPH) [13]. The isoflavonoids consist of three subgroups. The isoflavones exhibited the highest antioxidant potency based on all three assays. The additional presence of an OH in ring B at either R3′ or R5′ from the basic structure of R7-OH in ring A, and R4′-OH or -OMe of ring B increased the antioxidant activities of all isoflavonoid subgroups.

Modeling via QSAR became important in the drug candidate (new chemical entity, NCE) design, environmental fate modeling, toxicity and property prediction of chemicals, since they offer an economical and time-effective alternative to the medium-throughput *in vitro* and low-throughput *in vivo* assays [14,15]. A QSAR model is a simple mathematical equation, which is evaluated from a set of molecules with known activities, properties and toxicities via computational approaches. Hypothesis of QSAR supports the replacement, refinement and reduction (3Rs) in animals in the research paradigm as an alternative for untested NCEs [16]. Tropsha and co-workers reviewed QSAR [17]. A QSAR model is limited to query chemicals structurally similar to the training compounds in the applicability domain (AD). Robust validation of QSAR relationships is key for a predictive model, which may be considered for forecasting molecules via interpolation (true prediction) inside AD or extrapolation (less reliable guess) outside AD. A test molecule that is similar to those in the training set is predicted by QSAR model developed on the corresponding training set. On the contrary, a molecule quite dissimilar to the training ones will never be predicted with the same efficacy, since it is impossible for a single QSAR model to capture the property of an entire universe of chemicals. Relationships of QSAR present applications in drug discovery, environmental fate modeling, risk assessment and chemicals property prediction. The addition of descriptors to a model leads to a rise in the correlation coefficient but this does not always indicate an improvement in predictability. Models of QSAR were used for developing drugs. An objective of QSAR modeling is to predict absorption, distribution, metabolism, excretion (ADME), activity, property and toxicity (ADMET) of NCEs falling within developed-models AD. Chemical qualification (QSAR) programs depend on quantification of physicochemical and physiochemical properties, which facilitate selectivity towards antioxidant capacity.

In earlier publications, quantitative structure-property relationships (QSPRs) allowed prediction of chromatographic retention times of phenylurea herbicides [18] and pesticides [19]. This study aimed to investigate isoflavonoids QSARs via X/XO (pH 9.4), ORAC (blood-serum physiological pH 7.4) and DPPH (methanol, MeOH) assays via different solvents: inhibitions of water-soluble superoxide radical O_2_^•−^ formation and peroxyl radical HO_2_^•^-induced oxidation, and water-insoluble DPPH, respectively. Antioxidant capacities were derived from Promden *et al*. [10]. The improvements with regard to this qualitative work have been illustrated and discussed. In our QSARs, the different activity trends for the three complementary assays are explained.

## 2. Results and Discussion

The molecular structures of 17 isoflavonoids, *viz*. eight isoflavones, six isoflavanones and three isoflavans, from the heartwood (duramen) of *D. parviflora* are displayed in Table 1. However, the obtained results are limited to the 17 substances contained in the ChEMBL database.

Isoflavonoids antioxidant activities in ORAC, X/XO and DPPH model assays were derived from Promden *et al*. [10]. However, no QSAR analysis was provided. For inactive Entries 12–14, 14 and 3–7–8–12–13–14 in Table 2, ORAC Trolox™ (a water-soluble vitamin-E analogue) equivalent antioxidant capacity (TEAC) was taken as minimum (minimum log ORAC), X/XO and DPPH concentration for 50% radical-trapping (*scavenging*, SC_50_) were taken as maximum. Notice the opposite trends of ORAC and X/XO-DPPH results.

**Table 1:** Molecular structure of isoflavonoids from *Dalbergia parviflora*.

**Molecular Structure**	**Entry**	**Isoflavones**	**R_5_**	**R_7_**	**R_2_′**	**R_3_′**	**R_4_′**	**R_5_′**
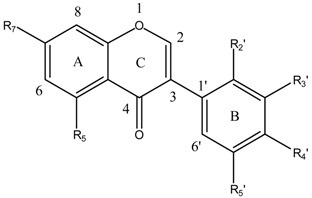	1	Khrinone C	OH	OH	OMe	OH	OMe	H
	2	Calycosin	H	OH	H	OH	OMe	H
	3	Genistein	OH	OH	H	H	OH	H
	4	3′-*O*-Methylorobol	OH	OH	H	OMe	OH	H
	5	Cajanin	OH	OMe	OH	H	OH	H
	6	Khrinone B	OH	OH	OH	H	OMe	OH
	7	Biochanin A	OH	OH	H	H	OMe	H
	8	Formononetin	H	OH	H	H	OMe	H
**Molecular Structure**	**Entry**	**Isoflavanones**	**R_5_**	**R_7_**	**R_2_′**	**R_3_′**	**R_4_′**	**R_5_′**
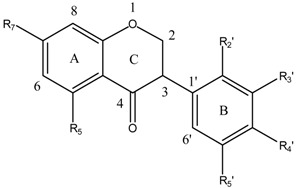	9	3(*R*,*S*)-Violanone	H	OH	OMe	OH	OMe	H
	10	3(*S*)-Secundiflorol H	OH	OH	OMe	OH	OMe	H
	11	3(*R*,*S*)-Dalparvin	H	OH	OMe	H	OMe	OH
	12	3(*R*,*S*)-Onogenin	H	OH	OMe	H	OCH_2_O	
	13	3(*S*)-Sativanone	H	OH	OMe	H	OMe	H
	14	3(*R*,*S*)-3′-*O*-Methylviolanone	H	OH	OMe	OMe	OMe	H
**Molecular Structure**	**Entry**	**Isoflavans**	**R_7_**	**R_8_**	**R_2_′**	**R_3_′**	**R_4_′**	**R_5_′**
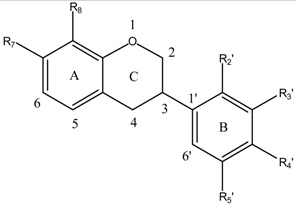	15	3(*R*)-Vestitol	OH	H	OH	H	OMe	H
	16	3(*R*)(+)-Mucronulatol	OH	H	OMe	OH	OMe	H
	17	3(*S*)-8-Demethylduartin	OH	OH	OMe	OH	OMe	H

**Table 2:** Antioxidant activity (X/XO, ORAC, DPPH assays) of isoflavonoids from *D. parviflora* and ChEMBL physico/physiochemical descriptors.

Entry	X/XO Assay SC_50_ [μM] ^a^	ORAC Assay TE [μM] ^b^	DPPH Assay SC_50_ [μM] ^a^	Log X/XO	Log ORAC	*M*w [Da] ^c^	ALog *P* ^d^	ACD Log *P* ^e^	ACD Log *D* ^f^	ACD p*K*_a_^g^	RBN ^h^	QEDw ^i^	PSA [Å^2^] ^j^	NH + OH	HBD ^k^	N + O	HBA ^l^
1	0.64	43.5	61.7	−0.194	1.638	330	2.11	2.25	0.82	6.32	3	0.79	105	3	3	7	7
2	0.25	37.8	96.2	−0.602	1.577	284	2.37	1.33	0.75	6.95	2	0.89	76	2	2	5	5
3	9.0	37.8	300	0.954	1.577	270	2.14	3.11	1.93	6.51	1	0.74	87	3	3	5	5
4	36.7	35.7	81.2	1.565	1.553	300	2.12	2.63	1.25	6.35	2	0.79	96.2	3	3	6	6
5	54.3	34.7	70.8	1.735	1.540	369	3.52	3.88	3.86	8.93	8	0.54	96.2	3	3	6	6
6	0.60	34.2	133.6	−0.222	1.534	316	1.88	1.71	0.37	6.38	2	0.63	116	4	4	7	7
7	203.3	26.6	300	2.308	1.425	284	2.37	3.34	2.11	6.5	2	0.89	76	2	2	5	5
8	116.92	2.8	300	2.068	0.447	268	2.61	6.99	2.86	2.31	2	0.91	55.8	1	1	4	4
9	43.7	31.1	89.7	1.640	1.493	286	2.48	7.69	2.63	2.44	2	0.89	76	2	2	6	5
10	247.2	27.4	74.3	2.393	1.438	302	2.24	2.76	2.34	7.5	2	0.79	96.2	3	3	7	6
11	48.2	21.8	80.4	1.683	1.338	332	2.22	7.48	3.01	2.58	3	0.79	105	2	3	6	7
12	56.9	0.0	300	1.755	0.0	330	2.25	4.52	4.1	7.48	2	0.87	94.4	1	2	6	7
13	59.3	0.0	300	1.773	0.0	270	2.72	3.48	3.31	7.7	2	0.91	55.8	1	1	5	4
14	300	0.0	300	2.477	0.0	330	2.69	2.93	2.74	7.67	7	0.93	74.2	1	1	6	6
15	6.4	40.1	204.1	0.806	1.603	272	3.2	3.26	3.25	9.53	2	0.88	58.9	2	2	4	4
16	10.0	39.8	75.41	1.000	1.600	302	3.18	2.84	2.84	9.87	3	0.91	68.2	2	2	5	5
17	13.4	27.0	115.4	1.127	1.431	318	2.94	1.65	1.65	9.75	3	0.75	88.4	3	3	6	6

^a^ SC_50_: concentration providing 50% inhibition; ^b^ Expressed as Trolox equivalents (TE, μM Trolox)/10 μM isoflavonoid; ^c^ *M*w: molecular weight; ^d^ ALog *P*: decimal logarithm of the 1-octanol-water partition coefficient (log *P*) calculated by the method ALog *P*; ^e^ ACD Log *P*: log *P* calculated by ACD/Log *P*; ^f^ ACD Log *D*: decimal logarithm of the 1-octanol-water distribution coefficient (log *D*) calculated by ACD/Log *D* at p*H* 7.4; ^g^ ACD Acidic p*K*_a_: p*K*_a_ calculated by ACD/p*K*_a_; ^h^ RBN: rotatable bonds; ^i^ QEDw: weighted quantitative estimate of drug-likeness; ^j^ PSA: topological polar surface area; ^k^ HBD: hydrogen-bond donor; ^l^ HBA: hydrogen-bond acceptor.

Isoflavonoids (IfOH) scavenge free radicals R^•^ according to three possible reducing pathways. (i) H-atom transfer (HAT) from the molecule to the radical (direct O–H bond breaking):

(1) $$\text{IfOH (antioxidant)}+{\text{R}}^{•}\text{(free radical)}\to {\text{IfO}}^{•}+\text{RH}$$

High HAT rate is expected for a low O–H bond dissociation enthalpy (BDE). (ii) Electron transfer (ET) from molecule to radical, leading to indirect H-abstraction or proton transfer (PT) (ET-PT):

(2) $$\text{IfOH (antioxidant)}+{\text{R}}^{•}\text{(free radical)}\to {\text{IfOH}}^{•+}+{\text{R}}^{\text{–}}\to {\text{IfO}}^{•}+\text{RH}$$

(iii) Sequential proton-loss-electron-transfer (SPLET). Since antioxidants primarily function by HAT, which involves formation of an H-bond with the harmful free radicals [20], a rise in the count of OH substituents facilitates interaction with the toxic radicals (Fujita-Ban analysis) [21].

### 2.1. Correlations between the Different Methods, and Physicochemical and Physiochemical Properties

Physicochemical and physiochemical properties of isoflavonoids were calculated (NH + OH, N + O) or taken from ChEMBL database: *steric* (molecular weight, *M*w), lipophilic (log *P*/*D*, topological polar surface area, PSA), acid (p*K*_a_), flexibility (rotatable bond, RBN), drug-likeness (weighted quantitative estimate of drug-likeness, QEDw, QED) and H-bond donor/acceptor (HBD/A) [22]. All *M*w < 400 Da were in agreement with the rule of five (RO5). Cajanin (Entry 5 in Table 2) *M*w = 369 Da and its log *P*/PSA could be decreased. All ACD log *P* < 5 according to RO5 with the exception of Entries 8, 9 and 11. However, these results should be taken with care because atom type summation log *P* (Alog *P*) < 3 and log *D* < 4. All log *D* = 0–3 predicting high oral bioavailability (OB) except Entries 5, 11–13 and 15. All p*K*_a_ = 2–10 and isoflavonoids are weak acids in water, most resulting anionic while they are neutral without separation of charges in organic solvents (MeOH). Entries 8, 9 and 11 present maximum ACD log *P* ~7 and minimum p*K*_a_ ~2. All RBN ≤ 8 and N + O ≤ 7 forecasting OB. All QED > 0.7 (highly drug-like, HD) except Entries 5 and 6: QED = 0.5–0.7 (drug-like, D). It decays with *M*w, *etc*. Entries 2, 3, 7, 8, 13, 15 and 16 with N + O = 4–5 present a chance of entering BBB. Entries 8, 13, 15 and 16 show PSA < 70 Å^2^, and are foreseen with OB and to penetrate BBB in agreement with N + O = 4–5. Entries 15 and 16 with Alog *P* > 3 when PSA < 70 Å^2^ carry toxicity risk. Entries 1–7, 9–12, 14 and 17 show 70 < PSA < 120 Å^2^ and are envisaged with high/middle OB. All NH + OH ≈ HBD ≤ 4 and N + O ≈ HBA ≤ 7 following RO5. The PSA trends are similar to HBA.

#### 2.1.1. Xanthine/Xanthine Oxidase Assay

Most isoflavonoids exhibited high antioxidant activity in X/XO assay. The role of ring C is confirmed in the presence of the 2,3-double bond. Fragment =O environment primarily dictates its contribution to the antioxidant capacity profile of isoflavonoids. The class of planar isoflavones showed the highest potency. The activity of the different divisions were confirmed comparing the capacity of compounds with the same substitution pattern: planar, ring-C-unsaturated isoflavone khrinone C was detected much more potent than nonplanar, ring-C-saturated isoflavan 3(*S*)-8-demethylduartin and isoflavanone 3(*S*)-secundiflorol H (Entries 1, 17 and 10, respectively). The X/XO correlated with PSA and HBD properties. Conversion of X/XO to its logarithm got a better relationship with log *D* and p*K*_a_ descriptors. The best linear fit turns out to be:

(3) $$-\text{Log X/XO}=-\left(0.494\pm 0.647\right)-(0.570\pm 0.167)\text{ACD Log }D+(0.0769\pm 0.0765)\text{ACD p}K_{\text{a}}\;n\text{ = }17\text{, }r\text{ = }0.683\text{, }s\text{ = }0.725\text{, }F\text{ = }6.1\text{, MAPE = }38.48\%\text{, AEV = }0.5340\text{, }q\text{ = }0.553$$

where *n* is the number of points, *s* standard deviation, *F* Fischer ratio, MAPE mean absolute percentage error, AEV approximation error variance and *q*, leave-1-out cross-validated (CV) correlation coefficient. The p*K*_a_ correlates positively, while log *D* associates negatively, with −log X/XO. The positive coefficient for p*K*_a_ implies that activity rises for weaker-acids isoflavonoids in agreement with the fact that the assay prefers isoflavans (p*K*_a_ ≈ 10) to isoflavanones (p*K*_a_ ~6). The negative coefficient for log *D* signifies that capacity rises for isoflavonoids more stable in the aqueous than in the organic phase. If a quadratic term is included in the fit, the model is improved:

(4) $$\begin{array}{l} -\text{Log X/XO}=\left(2.97\pm 1.31\right)-\left(2.44\pm 0.63\right)\text{ACD Log }D+\left(0.410\pm 0.136\right)\text{ACD (Log }D{\text{)}}^{2}\\ -\left(0.229\pm 0.156\right)\text{HBA} \end{array}\;n\text{ = }17\text{, }r\text{ = }0.833\text{, }s\text{ = }0.570\text{, }F\text{ = }9.8\text{, MAPE = }30.42\%\text{, AEV = }0.3104\text{, }q\text{ = }0.726$$

and AEV decays by 42%. Log *D* correlates negatively with −log X/XO in agreement with Equation (3). However, (log *D*)^2^ correlates positively with −log X/XO in a model passing via a minimum, in agreement with log *P* parabolic models of *in vitro* penetration of xenobiotics across artificial lipoidal/biomembranes [23]. Its small absolute coefficient indicates a weak nonlinear relationship. Linear Equation (3) has only two variables and is better appropriated for extrapolation than nonlinear Equation (4).

#### 2.1.2. Oxygen Radical Absorbance Capacity Assay

Most isoflavones showed high antioxidant activity in ORAC assay, which correlated with PSA and HBD properties. The conversion of ORAC to its logarithm got better relationship with the same descriptors. The best linear fit results:

(5) Log ORAC=(1.37±0.49)−(0.0335±0.0101)PSA+(1.12±0.21)HBDn = 17, r = 0.842, s = 0.361, F = 17.1, MAPE = 22.58%, AEV = 0.2903, q = 0.758

The HBD ≈ NH + OH correlates positively with log ORAC in agreement with Fujita-Ban analysis. However, PSA associates negatively with log ORAC. Adding two quadratic terms, fit is improved:

(6) $$\begin{array}{l} \text{Log ORAC}=-\left(0.966\pm 0.455\right)+\left(0.00435\pm 0.00268\right)\text{ACD Log }P^{2}\\ -\left(0.0975\pm 0.0536\right)\text{ACD Log }D-\left(0.122\pm 0.060\right)\text{N + O +}\left(2.40\pm 0.25\right)\text{NH + OH}\\ \;-\left(0.411\pm 0.057\right){\text{ (NH + OH)}}^{2} \end{array}\;n\text{ = }17\text{, }r\text{ = }0.972\text{, }s\text{ = }0.177\text{, }F\text{ = }38.1\text{, MAPE = }9.37\%\text{, AEV = }0.0600\text{, }q\text{ = }0.870$$

and AEV decays by 79%. The NH + OH ≈ HBD correlates positively with log ORAC in agreement with Fujita-Ban analysis and Equation (5). However, log *D* and N + O associate negatively with log ORAC. Quadratic ACD log *P*^2^ correlates positively with log ORAC in a parabola with a minimum, while (NH + OH)^2^ associates negatively in a parabola with a maximum. Linear Equation (5), with only two variables, results better suited for extrapolation than nonlinear Equation (6).

#### 2.1.3. 2,2-Diphenyl-1-picrylhydrazyl Assay

Most isoflavones displayed high antioxidant activity in DPPH assay, which correlated with properties Alog *P*, QED, N + O and HBD. Best linear fit is:

(7) $$\begin{array}{l} -\text{DPPH}=-\left(1390\pm 513\right)+\left(134\pm 54\right)\text{ALog }P+\left(458\pm 347\right)\text{QED}+\left(40.2\pm 27.2\right)\text{N}+\text{O}\\ \;+\left(120\pm 47\right)\text{HBD} \end{array}\;n\text{ = }17\text{, }r\text{ = }0.790\text{, }s\text{ = }73.881\text{, }F\text{ = }5.0\text{, MAPE = }32.53\%\text{, AEV = }0.3938\text{, }q\text{ = }0.400$$

All descriptors correlate positively with −DPPH, and HBD ≈ NH + OH is in agreement with Fujita-Ban analysis and Equations (5) and (6). A positive coefficient for log *P* implies that antioxidant activity in the assay rises for isoflavonoids more soluble in the organic than in the aqueous phase. As DPPH assay is in MeOH (not water), the corresponding interpretation is that water, compared to MeOH, presents the capacity of forming a number of H-bonds (nets), while MeOH affinity for creating H-bonds is smaller because of the *steric* interference of the CH_3_ group and inability to receive-give more H atoms. This is in concordance with the positive sign of log *P* and N + O terms. If quadratic p*K*_a_^2^ is included in the fit, the correlation is improved:

(8) $$\begin{array}{l} -\text{DPPH}=-\left(2090\pm 501\right)+\left(235\pm 68\right)\text{ALog }P-\left(1.58\pm 0.83\right)\text{ACD p}{K_{\text{a}}}^{2}+\left(800\pm 317\right)\text{QED}\\ \;+\left(70.1\pm 23.6\right)\text{N}+\text{O}+\left(151\pm 44\right)\text{HBD} \end{array}\;n\text{ = }17\text{, }r\text{ = }0.865\text{, }s\text{ = }63.201\text{, }F\text{ = }6.5\text{, MAPE = }22.79\%\text{, AEV = }0.2693\text{, }q\text{ = }0.655$$

and AEV decays by 32%. All linear descriptors correlate positively with DPPH in agreement with Equation (7), and HBD ≈ NH + OH is in concordance with Fujita-Ban analysis. Quadratic p*K*_a_^2^ associates negatively with −DPPH in a parabolic model with a maximum. Linear Equation (7) with only four variables is better appropriated for extrapolation than nonlinear Equation (8). The use of log DPPH as dependent variable does not improve the models.

#### 2.1.4. Comparison between the Three Methods

The log X/XO can be estimated from log ORAC:

(9) Log X/XO=2.18−0.729Log ORAC

The log X/XO can be approximated from DPPH:

(10) Log X/XO=0.721+0.00347DPPH

The DPPH can be calculated from log ORAC:

(11) DPPH=(308±41)−(117±31)Log ORACn = 17, r = 0.702, s = 76.797, F = 14.6, q = 0.638

in agreement with the opposite trends of X/XO-DPPH and ORAC. The correlation is poor (Equation (11)). However, when a correction is made for the fact that ORAC assay is in water while DPPH assay is in MeOH, by adding a term in log N + O, a better fit is obtained:

(12) DPPH=(725±164)−(107±26)Log ORAC−(574±221)Log N+On = 17, r = 0.811, s = 65.310, F = 13.4, q = 0.748

where the term in log N + O ≈ log HBA corrects for the fact that in the ORAC assay, water presents greater ability to H-bond transfer than MeOH in the DPPH test.

The physicochemical and physiochemical properties used in Table 2 are simple to calculate, and their use gained widespread acceptance but the bulk physical properties of molecules are correlated [24]. One issue in using these properties is the potential redundancy, which is illustrated simply among isoflavonoids, where all four RO5 parameters are clearly linked:

(13) $$\begin{array}{l} \text{ALog }P=-\left(0.418\pm 0.540\right)+\left(0.0219\pm 0.0027\right)\text{MW}-\left(0.697\pm 0.086\right)\text{HBA}\\ \;+\left(0.0745\pm 0.0704\right)\text{HBD} \end{array}\;n\text{ = }17\text{, }r\text{ = }0.934\text{, }s\text{ = }0.181\text{, }F\text{ = }29.5\text{, }q\text{ = }0.824$$

in agreement with the data for oral drugs taken from literature (N + O ≈ HBA, NH + OH ≈ HBD) [25]:

(14) CLog P=0.19+0.018MW−0.64N+O−0.40NH+OHn = 1193, r = 0.79

The standard errors of the coefficients show that all ones in Equations (3)–(13) are acceptable.

Leave*-m-*out (1 ≤ *m* ≤ 14) CV correlation coefficient *r*_cv_ calculated for isoflavonoids (*q* = *r*_cv_ (*m* = 1), *cf*. Table 3) show that *r*_cv_ decays with *m* except −DPPH (Equation (7)) and Alog *P* (Equation (13)), which indicate possible outliers. In particular, both antioxidant activity models log ORAC *vs*. {PSA, HBD} (Equation (5)) and *vs*. {(ACD log *P*)^2^, log *D*, N + O, NH + OH, (NH + OH)^2^} (Equation (6)) give the greatest *r*_cv_. The interpretation is that these are the most predictive descriptors sets for modeling isoflavoniods antioxidant activity. However, models −log X/XO *vs*. {log *D*, (log *D*)^2^, HBA} (Equation (4)) and −DDPH *vs*. {Alog *P*, (*p*K_a_)^2^, QED, N + O, HBD} (Equation (8)) give smaller *r*_cv_. Equation (6) is more predictive than Equations (4) and (8).

Drug design, discovery and development are complex and difficult because drug action is much more than binding affinity. A successful, efficacious and safe drug must present a balance of properties, e.g., activity against its intended target, appropriate ADME and acceptable safety profile. Based on the obtained results, new definitions of (stringent) drug-likeness, tractability and central nervous system (CNS)-active are proposed. Drug-likeness evaluates the suitability of the molecule under RO5, *etc*. The CNS-active is stricter. However, tractability is under more relaxed conditions. A summary of physicochemical and physiochemical descriptors was selected for every property (*cf*. Table 4). Properties *M*w, Clog *P* (estimated as Alog *P*), RBN, QEDw, N + O, NH + OH, HBD, HBA, and no metal, sugar and carbohydrates fulfill drug-likeness for all 17 isoflavonoids. The only exception is khrinone B, which presents a Clog *P* − (N + O) ≈ Alog *P* − (N + O) = −5.12 ≤ −5 and PSA = 116 > 105 Å^2^ but it fulfills tractability: Alog *P* − (N + O) > −8 and PSA ≤ 140Å^2^.

**Table 3:** Cross-validation correlation coefficient in a leave*-m-*out procedure for isoflavonoids.

*m*	−Log X/XO Equation (3)	−Log X/XO Equation (4)	Log ORAC Equation (5)	Log ORAC Equation (6)	−DPPH Equation (7)	−DPPH Equation (8)	DPPH Equation (11)	DPPH Equation (12)	ALog *P* Equation (13)
1	0.553	0.726	0.758	0.870	0.400	0.655	0.638	0.748	0.824
2	0.552	0.725	0.757	0.870	0.405	0.653	0.638	0.748	0.828
3	0.550	0.724	0.756	0.869	0.409	0.651	0.638	0.747	0.832
4	0.549	0.722	0.755	0.867	0.415	0.648	0.638	0.746	0.836
5	0.546	0.720	0.753	-	0.422	0.645	0.637	0.745	0.839
6	0.544	0.717	0.751	-	0.431	0.641	0.637	0.744	-
7	0.540	0.713	0.749	-	0.442	0.636	0.635	0.743	-
8	0.537	0.707	0.746	-	-	-	0.633	0.742	-
9	0.532	0.698	-	-	-	-	0.629	0.741	-
10	0.527	0.682	-	-	-	-	0.624	0.740	-
11	0.521	0.650	-	-	-	-	0.615	0.741	-
12	0.516	-	-	-	-	-	0.600	-	-
13	0.509	-	-	-	-	-	0.565	-	-
14	0.436	-	-	-	-	-	-	-	-

**Table 4:** Summary of physicochemical and physiochemical descriptors selected for every property.

Property	*M*w [Da] ^a^	CLog *P* ^b^	RBN ^c^	QEDw ^d^	N + O	CLog *P* − (N + O)	NH + OH	PSA [Å^2^] ^e^	HBD ^f^	HBA ^g^	Others
Tractability	200–800 ^i^	≤8 ^i^	≤16	>0.2	≤16	>−8	≤8	≤140	≤8	≤15	No metal, sugar, carbohydrates
Drug-likeness	100–500 ^i^	≤5 ^i^	≤10	>0.5	≤10	>−5	≤5	≤105	≤5	≤10	No metal, sugar, carbohydrates
Stringent drug-likeness	100–450 ^i^	≤4 ^i^	≤10	>0.5	≤8–9	>−4.5	≤3	≤105	≤3	≤8–9	No metal, sugar, carbohydrates
CNS-active ^h^	100–400	≤3.5 ^i,j^	≤7	>0.7	≤5	>0	≤4	≤70^j^	≤4	≤5	No metal, sugar, carbohydrates

^a^ *M*w: molecular weight; ^b^ CLog *P*: decimal logarithm of 1-octanol-water partition coefficient (log *P*) calculated by CLog *P*; ^c^ RBN: rotatable bonds; ^d^ QEDw: weighted quantitative estimate of drug-likeness; ^e^ PSA: topological polar surface area; ^f^ HBD: hydrogen-bond donor; ^g^ HBA: hydrogen-bond acceptor; ^h^ Central nervous system (CNS)-active: penetrating the blood-brain barrier (BBB); ^i^ *M*w > 400 Da when CLog *P* > 4 carries toxicity risk; ^j^ CLog *P* > 3 when PSA < 75 Å^2^ carries toxicity and promiscuity risks.

### 2.2. Discussion

This study is in agreement with Promden *et al*. [10], providing an extension and further discussion. It would be expected that the results of the present work had not change if the larger set of 24 compounds were considered. However, the obtained results are limited to the 17 substances contained in the ChEMBL database. The novelty finding in comparison to Promden *et al*. [10] is described in the following paragraphs, essentially: in the present study, a comparative analysis of the three assays in different solvents and pHs is illustrated and analyzed. The main difference is that the work of Promden *et al*. [10] is qualitative SAR while this study is QSAR. A possibility exists of integrating parameters sets but the structural data of Promden *et al*. would be only indicators of functional-groups absence/presence. The predictability of the approach would be qualitative but not quantitatively improved.

There are two main types of empirical QSAR models: linear models and nonlinear ones. The linear models provide an appropriate representation of the activity in a small neighborhood of a set of molecular properties. However, when the molecules are tried outside this constrained region, the model predictions will not be accurate. On the other hand, the quadratic models tend to capture more precisely the capacity behavior, making the adequate for predicting a real potency in a wide region of properties. Weak nonlinear relationships were detected between some physicochemical and physiochemical properties, especially log *P*, and isoflavonoids antioxidant activity in X/XO, ORAC and DPPH assays. Key strengths of the obtained descriptors follow: (1) easy to understand and apply; (2) compounds with *non-drug-like* properties lie in the regions of property space with poor precedence; and (3) good guide to avoid potential pitfalls.

Considering the structure of isoflavonoids, some parameters {log *P*, log *D*, PSA, HBD} are used. A simple linear correlation is proved to be a good model for the antioxidant activity of the molecules; other properties are redundant information. Procedure CV leave*-m-*out shows that {PSA, HBD} and {(ACD log *P*)^2^, ACD log *D*, N + O, NH + OH, (NH + OH)^2^} are the most predictive sets of descriptors for linear and nonlinear modeling isoflavonoids antioxidant capacity, respectively, according to the criterion of maximization of CV correlation coefficient. Both sets contain the essential characters of the antioxidant potency for isoflavonoid structures. The proposed method allows rapid estimation of the antioxidant activity for these molecules. The linear methods require that fewer parameters be estimated and, therefore, may be more parsimonious (Occam’s razor). Linear and nonlinear correlation models were obtained for isoflavonoids antioxidant capacity, pointing, not only to a homogeneous molecular structure of these molecules, but also to the ability to predict and tailor drug properties. The latter is nontrivial in pharmacology.

## 3. Experimental Section

The 1-octanol-water *partition coefficient P* is the ratio of concentrations of compound S:

(15) $$P=\frac{{\left[\text{S}\right]}_{\text{1–noctanol}}}{{\left[\text{S}\right]}_{\text{water}}}$$

Its decimal logarithm log *P* measures lipophilicity. The ALog *P* is calculated from a regression based on the hydrophobicity contribution of 115 atom {H, B-F, Si-Cl, Se-Br, I} kinds [26]. Every atom in every structure is classified into one of 115 sorts. Log *P* results:

(16) $$\text{Alog }P=\sum_{i}n_{i}a_{i}$$

where *n_i_* is the number of the atoms of type *i* and *a_i_* is hydrophobicity constant. Codes ACD/Log *P* and calculated log *P* (CLog P) [27] predict it from structure.

*Distribution coefficient D* is the ratio of sum of the concentrations of all forms of compound (unionized/ionized) in each phase; e.g., for a weak acid HA:

(17) $$D=\frac{{\left[HA\right]}_{\text{1–noctanol}}}{{\left[A^{–}\right]}_{\text{water}}+{\left[HA\right]}_{\text{water}}}$$

As log*D* is p*H* dependent, aqueous phase pH is buffered, e.g., blood-serum physiological pH 7.4 in ORAC assay. For unionizable compounds, log *P* = log *D*. 0 < log *D* < 3 enhances OB [28]. Code ACD/Log *D* predicts it understanding ionizable-molecules lipophilicity from structure. Programs ACD/Log *P − D* are modules of ACD/Percepta (ACD/Labs).

An *acid dissociation constant K*_a_ measures the strength of an acid in solution. It is the equilibrium constant for acid-base dissociation reaction. The larger *K*_a_, the more there is dissociation of the molecules in solution. Acids and neutrals present decreased toxicity risks related to bases [29]. Code ACD/p*K*_a_ predicts dissociation constants from structure.

An RBN is any single non-ring bond, bounded to nonterminal *heavy* (non-H) atom. Amide C–N bonds are not considered because of their rotational energy barrier. The count of RBNs measures the molecular flexibility.

An H atom attached to a relatively electronegative (EN) atom is an HBD [30]. The EN atom usually ranges from N to F atoms. The count NH + OH ≈ HBD. An EN atom, e.g., N to F atoms, is an HBA, whether it is bonded to an H atom or not (e.g., HBD ethanol presents an H atom bonded to an O atom, HBA O atom in diethyl ether does not show an H atom bonded to it). The count N + O ≈ HBA. The solvatochromic parameters are: dipolarity-polarizability π*, HBD acidity α and HBA basicity β [31].

The PSA of an organic is calculated by Ertl *et al*. method as a sum of fragment contributions [32]. The N/O-centered polar fragments are considered [33]. The PSAs are similar to HBA trends. The PSA describes drug absorption (e.g., OB, human carcinoma of colon cell line type-2 (Caco-2) permeability, BBB penetration). In order to enter BBB, most CNS drugs show PSA ≤ 70 Å^2^ but PSA ≤ 75 Å^2^ when Clog *P* > 3 carries toxicity and promiscuity risks [34]. When *M*w > 400 Da, Clog *P* > 4 presents some toxicity risk [35].

The QED combines eight characteristics: *M*w, ALog *P*, HBD/A, PSA, RBN, number of aromatic rings (AROM), and count of alerts for undesirable substructures (ALERT) [36]. It avoids the pitfalls of hard cut-offs, providing a single metric for *similarity* of a compound to known oral drugs [37]. Based on QED, molecules can be classified: nondrug-like (ND), poorly drug-like (PD), D and HD for QED in 0.0–0.2, 0.2–0.5, 0.5–0.7 and 0.7–1.0, respectively. The RO5 predicts OB when HBD ≤ 5, HBA ≤ 10, *M*w ≤ 500 Da and log *P* ≤ 5 [38]. Most OB compounds present RBN ≤ 10 and PSA ≤ 140 Å^2^ [39]. Drugs with OB show N + O ≤ 10. Rules predict CNS activity: (1) if N + O ≤ 5, the molecule presents a high chance of entering BBB; (2) if log *P* − (N + O) > 0, the compound is CNS-active [40]. The *M*w, log *P* and PSA decline with *M*w > 340 Da [41].

The correlation coefficient between CV representatives and the property values *r*_cv_ has been calculated with the leave-*m-*out procedure [42]. The process furnishes a new method for selecting the best set of descriptors: leave-*m-*out selects the best set of descriptors according to the criterion of maximization of the value of *r*_cv_.

The statistics *r*, *s* and *F* were calculated with Microsoft Excel (Microsoft Office 2015); MAPE and AEV were computed with Knowledge Miner Insights for Excel; CV correlation coefficients (*q*, *etc*.) were evaluated with leave*-m-*out [42].

## 4. Conclusions

From the present results and discussion, the following conclusions can be drawn.

1. Seventeen isoflavonoids from *Dalbergia* were selected from ChEMBL database representing *medicinal chemistry* compounds. Most are detected highly drug-like. Binary rules for compounds selection result risky: filters neglect valuable opportunities. Structure-antioxidant activity analyses indicate most important properties: log *D-*p*K*_a_, PSA-HBD and log *P-*QED-N + O-HBD for X/XO, ORAC and DPPH assays, respectively. Capacity in X/XO prefers weaker-acids isoflavonoids more soluble in water than in 1-octanol, in agreement with X/XO (pH 9.4) favoring neutral isoflavans (p*K*_a_ ≈ 10) rather than anionic isoflavanones (p*K*_a_ ~6). However, DPPH chooses isoflavonoids more soluble in 1-octanol with greater N + O count because this test is in methanol with H-bond transfer ability smaller than water. Models of QSAR provide quantitative information that filters drugs based on log *D*, *etc*. suggesting strategies for priority. Some descriptors (PSA, HBD) are more important than others (size, HBA). An advantage of our QSARs is that they detect weak nonlinear relationships between log *P*, *etc*. and potency. Simple, consistent analyses are described, improving our general understanding of activity. The rules are consistent with the literature.

2. Isoflavonoid ring-C role was confirmed in the presence of isoflavones 2,3-double bond, explaining their greatest activity. Capacity gave preferences: Planar unsaturated isoflavones greater than non-planar saturated isoflavans and isoflavanones because unsaturation and planarity stabilize the phenoxyl radical. On comparing isoflavanones with isoflavans, this study demonstrates different favorites of X/XO, ORAC and DPPH: X/XO (pH 9.4) prefers neutral isoflavans (p*K*_a_ ≈ 10) liking better phenoxyl-radical stabilization, which is not the case of anionic isoflavanones (p*K*_a_ ~6); in DPPH (methanol), an intramolecular H-bond R_4_ = O…HO-R_5_ can be formed in isoflavanones, but not in isoflavans lacking this moiety; and ORAC (pH 7.4) liking is intermediate. Isoflavonoids potency depends on the chemical form determining its solubility, which is modified by changing pH or solvent. Models of QSAR may predict activity of new series of isoflavonoids and design strong drugs.
